# Usefulness of Parent-Completed ASQ for Neurodevelopmental Screening of Preterm Children at Five Years of Age

**DOI:** 10.1371/journal.pone.0071925

**Published:** 2013-08-27

**Authors:** Marie Halbwachs, Jean-Baptiste Muller, Sylvie Nguyen The Tich, Elise de La Rochebrochard, Géraldine Gascoin, Bernard Branger, Valérie Rouger, Jean-Christophe Rozé, Cyril Flamant

**Affiliations:** 1 Department of Neonatal Medicine, Nantes University Hospital, Nantes, France; 2 Loire Infant Follow-up Team (LIFT) Network, Pays de Loire, France; 3 Department of Neonatal Medicine, Angers University Hospital, Angers, France; 4 INED, Paris, France; 5 INSERM, CESP, U 1018, Le Kremlin-Bicêtre, France; 6 Université Paris-Sud, UMRS 1018, Le Kremlin-Bicêtre, France; 7 National Institute of Health and Medical Research CIC004, Nantes University Hospital, Nantes, France; University of Tuebingen Medical School, Germany

## Abstract

**Introduction:**

Preterm children are at greater risk of developmental impairment and require close follow-up for early and optimal medical care. Our goal was to examine use of the parent-completed Ages and Stages Questionnaire (ASQ) as a screening tool for neurodevelopmental disabilities in preterm infants at five years of age.

**Patients and Methods:**

A total of 648 preterm children (<35 weeks gestational age) born between 2003 and 2004 and included in the regional Loire Infant Follow-up network were evaluated at five years of age. ASQ was compared with two validated tools (Intelligence Quotient and Global School Adaptation Score) and the impact of maternal education on the accuracy of this questionnaire was assessed.

**Results:**

Overall ASQ scores for predicting full-scale IQ<85 and GSA score produced an area under the receiver operating characteristic curve of 0.73±0.03 and 0.77±0.03, respectively. An ASQ cut-off value of 285 had optimal discriminatory power for identifying children with IQ scores<85 and GSA scores in the first quintile. ASQ values<285 were significantly associated with a higher risk of non-optimal neurologic outcomes (sensitivity of 0.80, specificity of 0.54 for IQ<85). ASQ values>285 were not distinctive for mild delay or normal development. In children with developmental delay, no difference was found when ASQ scores according to maternal education levels were analyzed.

**Conclusions:**

ASQ at five years is a simple and cost-effective tool that can detect severe developmental delay in preterm children regardless of maternal education level, while its capacity to identify children with mild delay appears to be more limited.

## Introduction

Neurodevelopmental impairment among preterm infants is a major concern [1]. The prevalence of cognitive disability and poor educational achievement is higher in this high-risk population, especially for those born very preterm [2]. These children need close follow-up for at least five years [3]–[4] to facilitate early detection of elements that predict non-optimal neurodevelopment. Their prompt identification is important as it can spur appropriate therapeutic interventions [5]. To assess the neurodevelopmental outcome of children born preterm, many formal standardized tests are available; these include the Kaufman Assessment Battery for Children (KABC) [6], the Wechsler Preschool and Primary Scale of Intelligence (WPPSI) [7], the Wechsler Intelligence Scale for Children (WISC) [8], and the Bayley Scales of Infant Development (BSID) [9]. The WPPSI is one of the most widely used psychometric assessment tools for measuring intelligence quotient (IQ). It has been used to explore the intellectual functioning of preschool children who underwent cardiac surgery [10], suffered from a severe neonatal illness [11], or were born preterm [12]–[13]. Although the full-scale IQ test constitutes the gold standard for evaluating children’s cognitive function, it can be regarded as inadequate because children born preterm can present disabilities in multiple domains such as speech, learning, behavior, cognition and attention, not all of which can be assessed with this instrument [14]. Moreover, this test should be performed by a trained psychologist and requires a lot of time because it is lengthy, making it difficult to perform during routine examinations.

Therefore, the current trend is to develop easier, simpler and less time-consuming tools for the preterm infant population in order to evaluate their neurodevelopmental outcome in its entirety. There is growing interest in monitoring the capacities of children born preterm with the support of people close to them, and new tools have been developed, such as questionnaires for parents and teachers [15]–[17]. These tools assess the child in his/her own environment, while standardized developmental tests like the IQ score are performed during a limited period, by persons unknown to the child, and are often tiring [18]. Screening tools completed by children’s proxies have been demonstrated to be of great value and to enable accurate and reliable assessments [19]–[21]. In this regard, the Global School Adaptation (GSA) score obtained from teacher questionnaires has been established as a reliable tool for the early detection of children with school adaptation difficulties that can result in learning disabilities [22]. A recent study found a good correlation between GSA and IQ scores at five years of age in children born preterm [23].

Several studies have shown the ability of parents to assess their child’s development [24]–[27]. The parent-completed Ages and Stages Questionnaires (ASQ) [28] involves five domains of development and has been found to be as reliable as standardized developmental tests [29]–[30]. Recently, the ASQ has been established as a valid tool to screen preterm-born children at two years of age, in comparison with the revised Brunet-Lezine psychometric test [31]. Nevertheless, to our knowledge, no study has examined the association between ASQ and formal psychometric assessments for preterm children at five years of age. Moreover, only two studies have used a parental questionnaire at 5 years of age and they were not designed to assess its usefulness [32]–[33].

The objective of the present study was to examine the value of the parent-completed ASQ as a screening tool for neurodevelopmental impairment in preterm children at five years of age. For this purpose, we compared ASQ scores with validated IQ and GSA scores and examined the effect of maternal education on the accuracy of the results obtained with this questionnaire.

## Patients and Methods

### Patients and Data Sources

All surviving children born ≤35 weeks of gestational age (GA) between January 2003 and December 2004 and enrolled in the regional “Loire Infant Follow-up Team” (LIFT) network program at discharge were included in the present study. Each child’s parents gave written informed consent before enrollment in the network. The network [34] includes 24 maternity hospitals, of which three are hospitals with neonatal intensive care units (Nantes, Angers, Le Mans). The patient database was registered with the French data protection authority for clinical research (Commission Nationale de l’Informatique et des Libertés CNIL, No. 851117). Specific approval to use the data in this study was obtained from the Institutional Review Board of the University of Nantes. Initial data were collected during hospitalization in the neonatal units. Regarding cerebral lesions, cranial ultrasound scans were regularly conducted for preterm infants born at less than 34 weeks of gestational age, according to the screening protocol established by Perlman JM et al [35] to identify severe intraventricular hemorrhage (i.e IVH 3–4) and periventricular leukomalacia. The children were further evaluated at 3, 9, 12, 18, 24, 36, 48 and 60 months of corrected age by trained pediatricians in our regional follow-up network. The children were then classified as possessing non-optimal neuromotor function when severe (resulting in a diagnosis of cerebral palsy with inability to walk independently) or moderate neuromotor impairment was present.

### Neurodevelopmental Assessment

The neurodevelopmental outcome of preterm-born children at five years of age was assessed with the WPPSI-III and the teacher-completed GSA questionnaires.

First, trained psychologists in our follow-up network evaluated the children with a French version of the standardized WPPSI-III test for children aged between four years and seven years and three months [7]. This test covers two major areas that are evaluated with two scales: verbal capacity and performance capacity. The verbal scale evaluates knowledge, verbal reasoning and comprehension, and attention to verbal stimuli; the performance scale evaluates fluid reasoning, spatial processing, attention to detail, and visual-motor integration. The child’s performance on these scales is used to compute a verbal intelligence quotient (Verbal IQ) and a performance intelligence quotient (Performance IQ). Next, the Full-Scale Intelligence Quotient (Full-scale IQ) is defined as the composite of verbal and performance IQ scores and is indicative of general intellectual functioning. The WPPSI-III psychometric assessment was constructed in order to have a mean full-scale IQ value of 100, with a standard deviation of 15. A full-scale IQ score lower than 85 was considered to define neurodevelopmental impairment and a full-scale IQ score lower than 70 was considered to define severe mental retardation.

The children were also assessed with the teacher-completed GSA questionnaire, which is considered as an educational tool for teachers, and includes 20 questions assessing linguistic competence, non-verbal abilities and children’s behavior in the classroom [23]. In the last part of the test, the teacher’s opinion of the child’s prognosis in terms of future school adaptation is obtained. Each item is scored from one to three, with three being the highest possible score for each item. The total score, defined as the sum of the individual item scores, ranges from 20 to 60. A score lower than 45 indicates that the child is likely to present difficulties later in school adaptation.

### Ages and Stages Questionnaire

The ASQ is a parent-completed screening test composed of 21 age-specific questions covering the age range 4 to 60 months [28]. In the present study, the 60-month questionnaire from the French translation of the second version was used. The French translation has been reviewed by a panel of French-speaking early childhood experts (Marthe Bonin Philippe Robaey, Sylvie Vandaele, Georges L. Bastin et Veronique Lacroix from the Canadian team) in collaboration with Diane Bricker and Jane Squires who established English ASQ version. It includes 30 developmental items divided into five domains of child capacities: communication abilities, gross motor skills, fine motor skills, problem solving abilities and personal-social skills. For each item, three responses are possible, depending on whether the child performs the task: “Yes” (10 points), “Sometimes” (5 points) and “Not Yet” (0 point). The total score for each domain is obtained by adding the scores of the six items. The overall ASQ score is established by combining the scores for the five domains, with a maximum global ASQ score of 300 points. Parents completed the ASQ between one month before and one month after the 60-month target age. The assessment usually requires approximately 15 minutes to complete. The ASQ was completed before the psychological assessment, so that the WPPSI test would not influence the parents’ evaluation. The pediatric psychologists in the regional network were blinded to the children’s ASQ results.

### Maternal Education

Levels of maternal education were assessed through a phone survey conducted by one of the network members. The data collected were computed into binary categorical variables (low vs. high). The level of maternal education was considered high if education continued for two years after obtaining a high school diploma.

### Statistical Analysis

Descriptive values were reported as medians and interquartile ranges for continuous variables, and numbers of subjects, frequencies and percentages for categorical variables. Sensitivity and specificity were expressed with a 95% confidence interval (CI). The significance threshold was set at p<0.05 for all analyses, which were performed using two-sided tests. GSA scores were expressed as quintiles: the first quintile corresponded to children with the worst results, and the fifth corresponded to those with the best results.

Neurodevelopmental impairment was defined as a full-scale IQ score lower than 85, or belonging to the first quintile of GSA scores, suggesting school difficulties. Crude associations between five-year global ASQ scores, full-scale IQ scores and GSA scores were also evaluated. Receiver operating characteristic (ROC) curves were generated to determine optimal cut-off values of ASQ scores, in terms of sensitivity and specificity, for the prediction of full-scale IQ scores lower than 85 and 70, and within the first quintile of GSA scores. Fisher’s exact test and Mann-Whitney test were used to assess the eventual effect of maternal education on ASQ scores. Z-statistics were used to compare the area under the curve (AUC) of the independent ROC curves, regardless of the level of maternal education. The statistical analyses were performed with SPSS v.15.0 (SPSS Inc.) and MedCalc v.11.5.1.

## Results

### Characteristics of the Study Population

**Figure 1:**
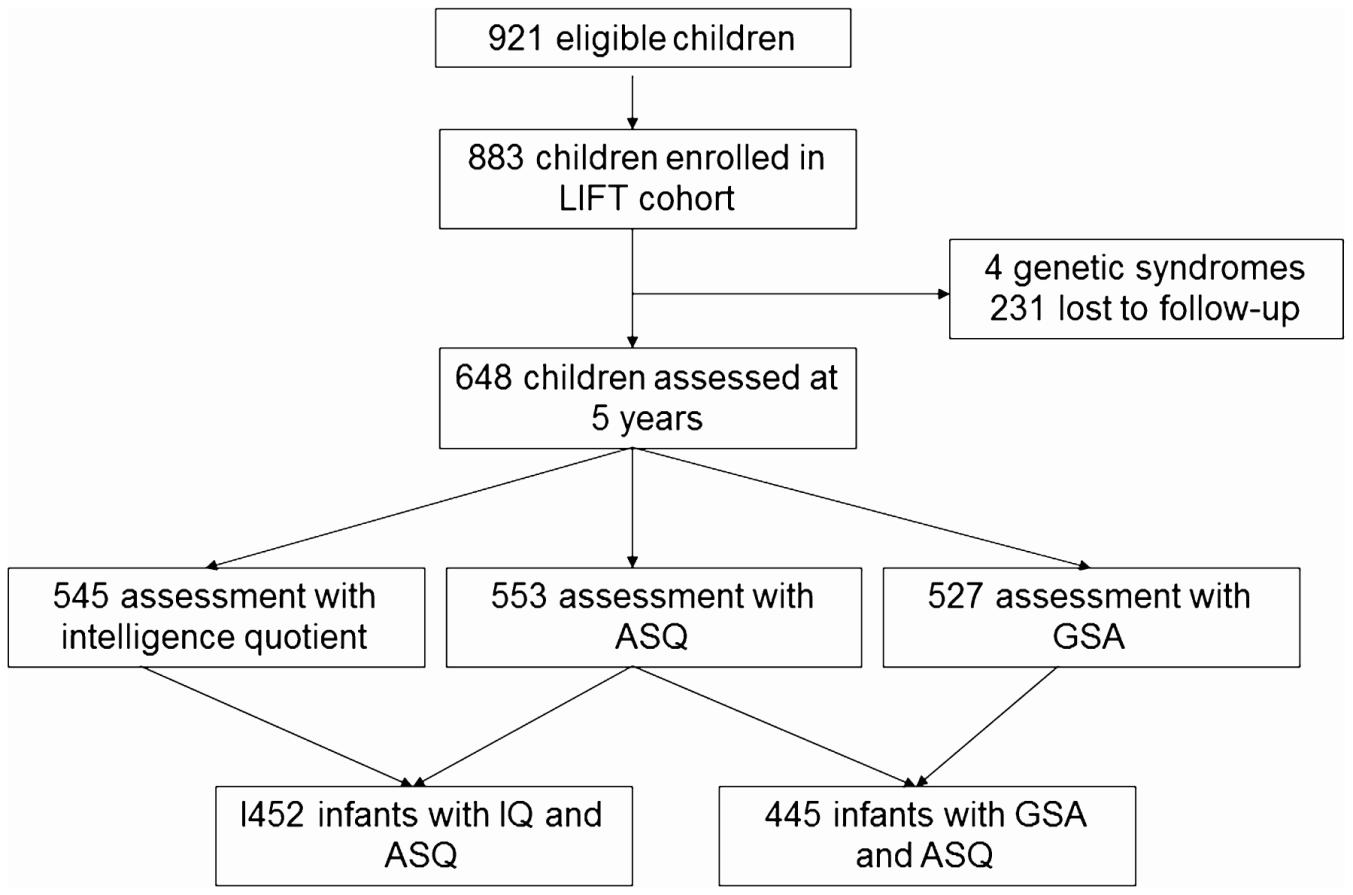
Cohort profile. LIFT: Loire Infant Follow-up Team; ASQ: Ages and Stages Questionnaire; GSA: Global School Adaptation.

Of the 921 children eligible at discharge, 883 (96%) were enrolled in the regional network. Among those, 648 children were assessed at five years of age (73%) (Figure 1). The characteristics of the study population are summarized in Table 1. No statistical difference in general characteristics was noted between the study group and the 235 children not assessed at five years, except for gestational age, as a higher number of very preterm children were assessed at 5 years. The subpopulation of premature infants who reached 5 years of age with non-optimal neuromotor function identified at 2 years of age was very small and included only 38 infants. Of these, 7 infants had ASQ evaluation and the mean ASQ score for these infants was 238. Detailed information on maternal education was available for 504 children.

**Table 1:** Characteristics of population enrolled in LIFT cohort (n = 883).

		Assessed at 5 years n = 648		Not assessed at 5 years n = 235			
		n	%	n	%		p
**Sex**							0.20
	Male	346	53.4	137	58.3		
	Female	302	46.6	98	41.7		
**Gestational age**							0.024
	23–27 wk	56	8.6	9	3.8		
	28–29 wk	57	8.8	28	11.9		
	30–31 wk	147	22.7	44	18.7		
	32–33 wk	229	35.3	81	34.5		
	34 wk	159	24.5	73	31.1		
**Birthweight**							0.85
	Unknown	18	2.8	9	3.8		
	<−2 SD	44	6.8	17	7.2		
	Between −2 and −1 SD	104	16	32	13.7		
	Between −1 and 1 SD	425	65.6	156	66.4		
	>1 SD	57	8.8	21	8.9		
**Cerebral lesions**							0.06
	Not assessed	150	23.1	55	23.4		
	No lesion	438	67.6	152	64.6		
	IVH 1–2	35	5.4	10	4.3		
	IVH 3–4 or PVL	25	3.9	18	7.7		
**Bronchopulmonary dysplasia**							0.10
	No oxygen	376	58	150	63.8		
	Oxygen<8 days	217	33.5	73	31.1		
	Oxygen<36 wk GA	38	5.9	5		2.1	
	Oxygen ≥36 wk GA	17	2.6	7		3	
		**n = 432**		**n = 72**			
**Maternal education**							
	High school diploma	209	48.4	33	45.8		0.61
	No high school diploma	223	51.6	39	54.2		

wk: weeks; SD: Standard Deviation; IVH: Intraventricular Hemorrhage; PVL: Periventricular leukomalacia; GA: Gestational Age.

### Primary Outcome at Five Years and ASQ Assessment

As indicated in Fig. 1, WPPSI-III scores were calculated for 545 children. However, full-scale IQ scores could be determined for only 452, because no coherence was found between verbal and performance ability in 93 children. The full-scale IQ score ranged from 41 to 140, with a median value of 96 [85–105]. A total of 91 children had full-scale IQ scores lower than 85 and 27 children had scores lower than 70. GSA scores were calculated for 527 children and ranged from 22 to 60, with a median value of 53 [45–57]; 92 children belonged to the first quintile, suggesting that they would experience school difficulties.

**Figure 2:**
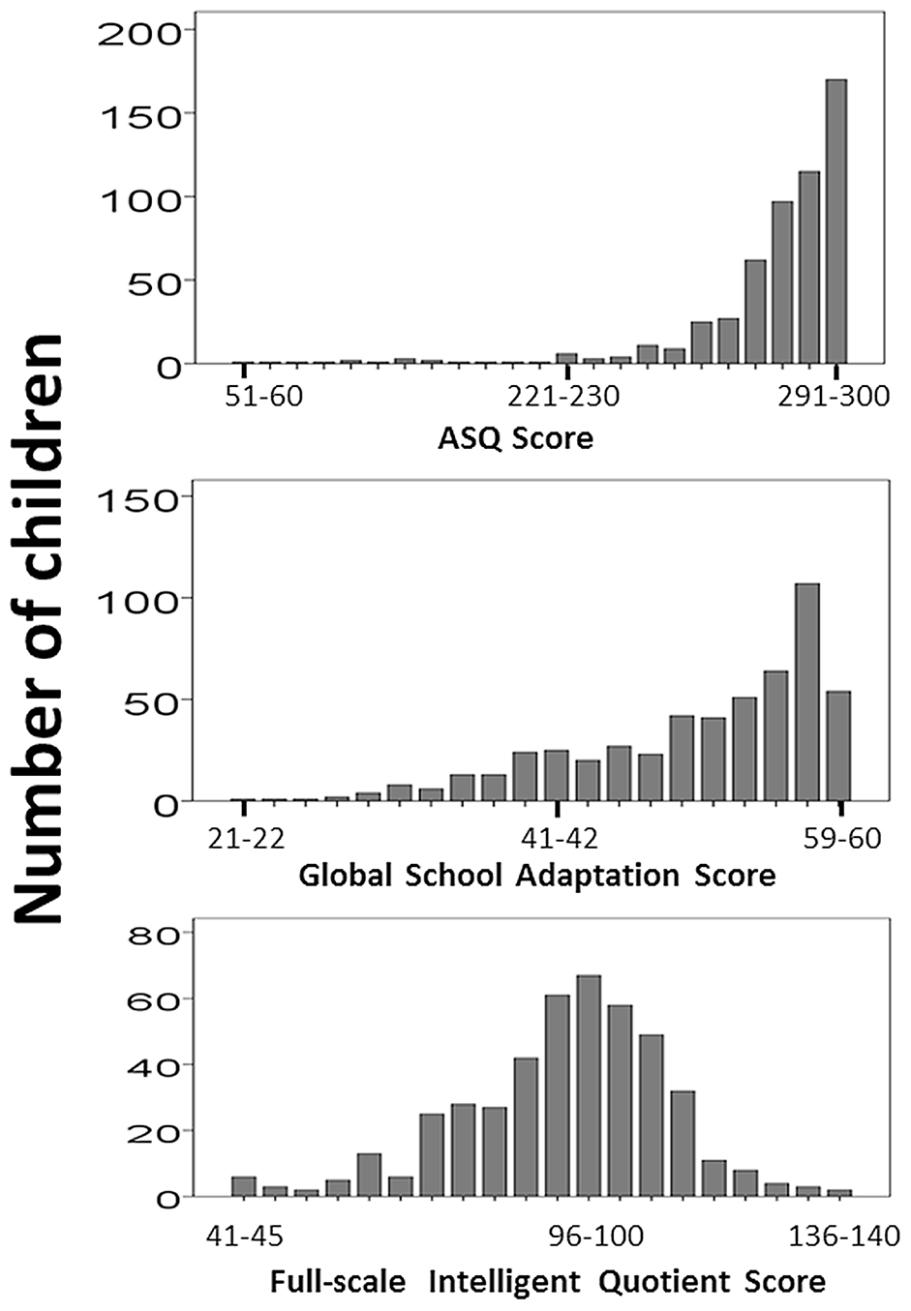
Distribution of global ASQ scores, GSA scores and full-scale IQ scores in study population.

ASQ scores were calculated for 553 children and global scores ranged from 60 to 300 with a median value of 285 [270–295]. The distributions of the scores for the three different assessments (full-scale IQ, GSA and ASQ) are presented in Fig. 2.

### ASQ and Full-scale IQ Scores

**Figure 3:**
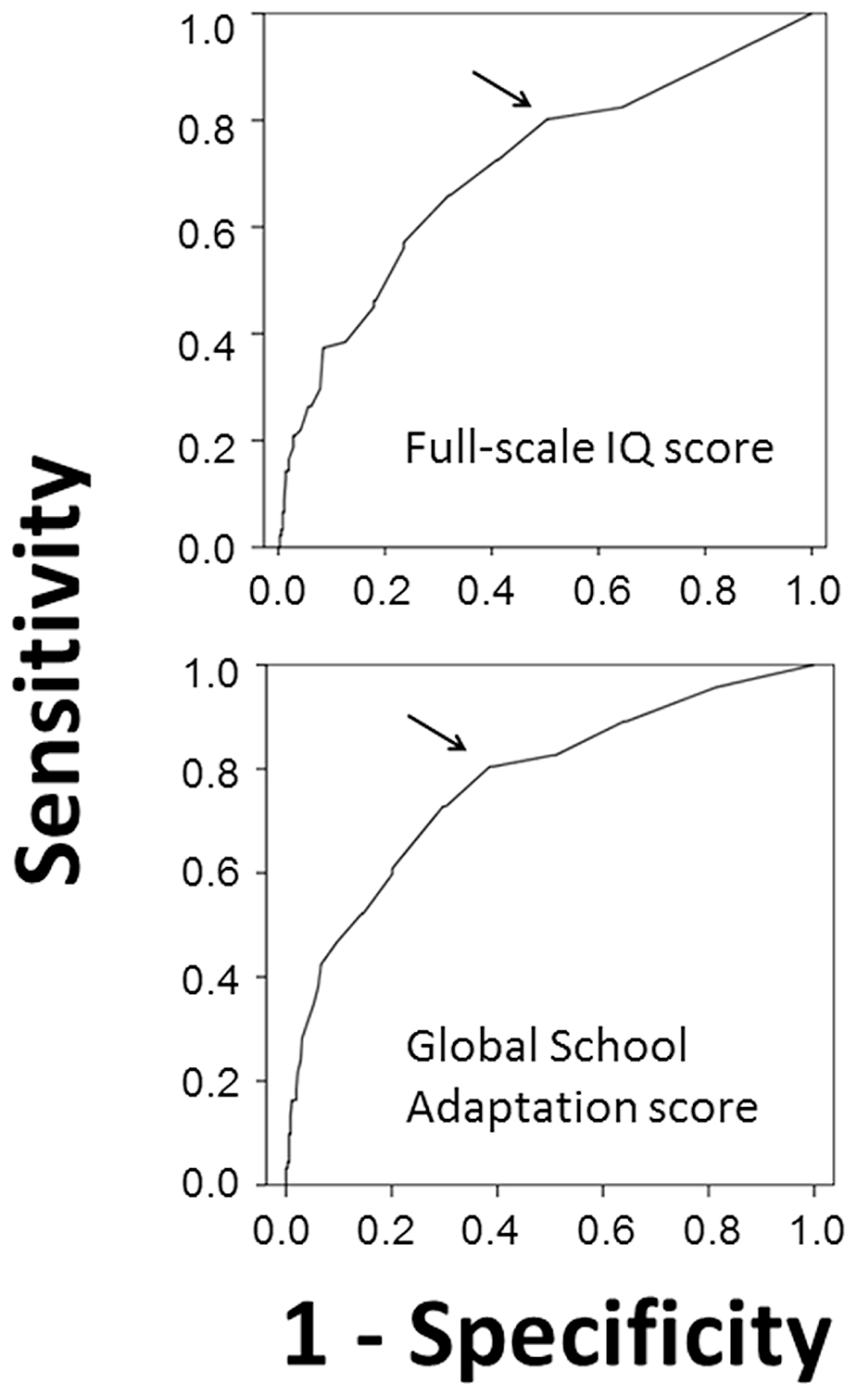
Receiver Operating Characteristic curves for predicting full-scale IQ score<85 and GSA score in first quintile based on ASQ values. Arrows denote optimal cut-off values (ASQ score of 285 for the two curves).

**Figure 4:**
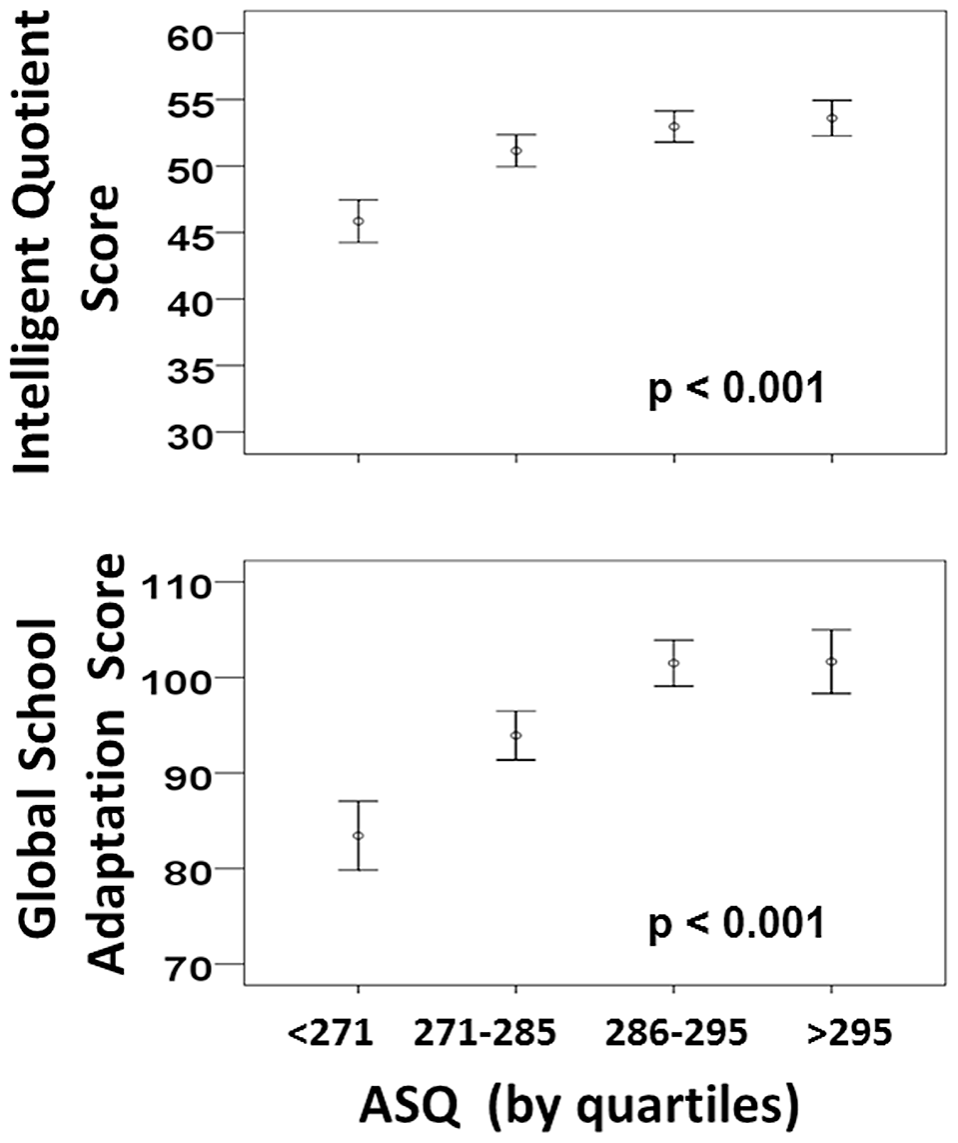
Correlations between global ASQ scores and full-scale IQ scores, and between global ASQ scores and GSA scores.

IQ scores and ASQ scores were calculated for 452 of the children assessed at five years of age. Using the overall ASQ score as a continuous variable, a ROC curve was generated to predict a full-scale IQ score lower than 85. ASQ scores produced an AUC value of 0.73±0.03. The optimal cut-off ASQ score value for identifying children with full-scale IQ scores<85 was 285, with a sensitivity of 0.80 (95% CI: 0.71–0.87) and a specificity of 0.54 (95% CI: 0.48–0.60) (Fig. 3). When a ROC curve was generated to predict a full-scale IQ score lower than 70, ASQ scores produced an AUC value of 0.90±0.04. The optimal cut-off ASQ score value was 270, with a sensitivity of 0.85 (95% CI: 0.68–0.94) and a specificity of 0.81 (95% CI: 0.77–0.85). Low ASQ scores correlated well with low full-scale IQ scores, but high values were not distinctive of normal or high full-scale IQ scores. The correlation between ASQ scores and full-scale IQ scores is shown in Fig. 4. (p<0.001).

Data on maternal educational was available for 308 out of 452 children: the mothers of 154 (50%) children had graduated from high school and the mothers of 154 (50%) children had not done so. When comparing the AUC corresponding to global ASQ scores in children with full-scale IQ scores lower than 85, no statistical difference was found according to the level of maternal education (had graduated from high school vs. had not graduated) (p = 0.72). Nevertheless, a global ASQ score of 285 predicted impairment with lower specificity in children whose mothers had not graduated from high school. The sensitivity was not significantly different, reaching 0.87 (95% CI: 0.70–0.95) in children whose mothers had not graduated from high school vs. 0.74 (95% CI: 0.51–0.88) in children in children whose mothers had done so. However, the specificity was significantly lower in children whose mothers had not graduated from high school (0.43; 95% CI: 0.32–0.55) than in children whose mothers had graduated from high school (0.67; 95% CI: 0.57–0.76). AUC values produced for global ASQ scores in children with a full-scale IQ score lower than 70 were higher than 0.90 regardless of the level of maternal education: the AUC was 0.90±0.04 in children whose mothers had not graduated from high school and 0.97±0.02 in children in children whose mothers had done so. A global ASQ score<270 showed a sensitivity of 0.80 (95% CI: 0.49–0.94) vs. 1.0 (95% CI: 0.57–1.00) and a specificity of 0.78 (95% CI: 0.68–0.85) vs. 0.89 (95% CI: 0.82–0.94) in children whose mothers had graduated from high school vs. children whose mothers had not done so.

### ASQ and GSA Scores

GSA and ASQ scores were calculated for 445 of the children assessed at five years of age. Using the overall ASQ score as a continuous variable, a ROC curve was generated to identify children in the first quintile of GSA scores. The AUC value produced by the overall ASQ score was of 0.77±0.03. The optimal cut-off ASQ value was 285, with a sensitivity of 0.82 (95% CI: 0.74–0.89) and a specificity of 0.50 (95% CI: 0.45–0.55) (Fig. 3). The correlation between ASQ and GSA scores is shown in Fig. 4 (p<0.001). Regarding full-scale IQ scores, ASQ scores<290 correlated well with GSA scores, although high values could not predict good school adaptation with certainty.

Data on maternal education were available for 321 children: the mothers of 155 (48%) children had graduated from high school and the mothers of 166 (52%) children had not graduated from high school. The AUC corresponding to global ASQ scores in children belonging to the first quintile of GSA score revealed no statistical difference in terms of the level of maternal education (had graduated from high school vs. had not graduated) (p = 0.47). Nevertheless, a global ASQ score of 285 predicted impairment with a lower specificity in children whose mothers had not graduated from high school 0.42 (95% CI: 0.34–0.51) compared with children whose mothers had done so 0.57 (95% CI: 0.48–0.65). The sensitivity was not significantly different, reaching 0.87 (95% CI: 0.74–0.94) in children whose mothers had not graduated from high school vs. 0.76 (95% CI: 0.53–0.91) in children whose mothers had done so.

## Discussion

This study, which was based on a large population of children born preterm, demonstrates that the parent-completed ASQ can predict severe developmental impairment at five years of age, and in particular can help to identify the most severely impaired children. An ASQ score<285 had fairly good sensitivity, but moderate specificity. This five-year ASQ threshold constitutes a useful screening instrument, regardless of the level of maternal education.

Global ASQ scores enable the detection of severe developmental delay but do not predict normal development if they are high. As previously described [31], we used the overall ASQ score as a continuous variable by combining the scores obtained for the five domains. This approach allowed us to determine an ASQ cut-off value of 285 to detect children with full-scale IQ scores<85 or children belonging to the first quintile of GSA scores. This cut-off ASQ value can be used to identify children who require a highly sensitive professional developmental assessment, thereby reducing the cost of full-scale outcome measurements. Thus, approximately 80% of children with developmental impairment were detected with the parent-completed report and approximately 70% of children with scores<285 actually had neurodevelopmental impairment. In contrast, the specificity of the test was not very high, but this point is not critically important, because the professional assessments are not invasive. The lack of specificity is probably due to the fact that the questionnaire administered at five years of age is based on very easy questions, unlike the questionnaire administered at two years of age, which predicts normal development with good accuracy in almost all children [31]. The results obtained here are more interesting because of the detection of severe developmental impairment (full-scale IQ score<70). Indeed, higher sensitivity and specificity were obtained for an ASQ cut-off value of 270, which corresponds to the 25^th^ percentile of the ASQ distribution in the study population. Nevertheless, the reproducibility and precision of this result remain unconfirmed, because the sample size was limited to 27 patients. In addition, we cannot extrapolate this result to children with normal or subnormal development. We showed that an overall ASQ score>290 did not always correlate with optimal neurodevelopment, even if the value was high. Thus, some children with mild neurodevelopmental impairment could have an overall ASQ score>290 and not be considered at risk. These mild issues are not always considered problematic [36], but have a significant influence on school performance, and these children may need early medical care to correct or attenuate problems [37]–[38].

In the present study, global ASQ results were not influenced by the level of maternal education, as previously described [31], [39]. However, other studies have shown that the level of maternal education was associated with the developmental outcome of children, especially in those born preterm [40]. Parental education can influence the assessment of child development [41] and may affect the reliability of parent-completed reports. It has been shown that mothers with higher educational achievements and those who are not working provide more accurate reports regarding their children’s development [42]. It is possible that mothers who did not graduate from high school had more difficulties in assessing clearly the development of their children, but this influence could be compensated for by the fact that mothers with higher educational achievements are more demanding and strict regarding their child’s abilities.

The main strength of our study was that a large number of children were included and monitored. Based on the substantial number of ASQ reports analyzed, we can support the validity and usefulness of the ASQ as a screening tool for detecting severe developmental impairment at five years of age in children born preterm. If neuromotor problems should be previously identified, ASQ is of particular interest for detecting cognitive delay in ex preterm infants who reach 5 years of age.

Nevertheless, 27% of the children in our study were not assessed at the age of five. These children presented similar neonatal characteristics to those of children assessed at five years, except for gestational age, but no information on their neurological outcomes were available. Our results indicate that the children assessed at the age of five were born more prematurely, but insufficient follow-up compliance was noted. Thus, use of the ASQ in studies with follow-up assessment of children could be very interesting, as it would enable easier access to assessment and follow-up procedures, especially for people with financial difficulties or those living in remote locations. The minor subpopulation of ex-premature infants with previously identified neurological difficulties (particularly motor and sensory problems) already received particular attention from intensive follow-up: the ASQ as a screening tool therefore seems less appropriate for them. A limitation of our study is that the French version of the 60-month-ASQ has not been validated in a control group. Nevertheless, previous studies have shown that the Dutch and Norwegian versions provide very similar results to those obtained with the original U.S. version [43]–[44]. These studies confirmed the cross-cultural validity of the ASQ, as only small differences were noted between the different European versions. They also confirmed the reliability of parent-completed reports for the neurodevelopmental assessment of children. Indeed, parents are highly involved in their children’s care and could be more receptive to the necessity to conduct follow-up assessments. Recently, Flamant et al. demonstrated that ASQ scores correlated with DQ scores obtained with the Brunet-Lezine scale at two years of age [31]. The findings of this study, which was also based on children enrolled in the LIFT cohort, led to modification of the network’s practices, in that the ASQ was administered to the parents of all children at two years of age, while the Brunet-Lezine scale was not systematically used.

In conclusion, our study demonstrates the usefulness of the ASQ for detecting severe developmental impairment in 5-year-old children born preterm, regardless of maternal education levels. Therefore, this questionnaire could be used to identify children at risk of developmental impairment who could benefit from receiving additional, more thorough assessments.
